# Benzodiazepines Associated With Acute Respiratory Failure in Patients With Obstructive Sleep Apnea

**DOI:** 10.3389/fphar.2018.01513

**Published:** 2019-01-07

**Authors:** Sheng-Huei Wang, Wei-Shan Chen, Shih-En Tang, Hung-Che Lin, Chung-Kan Peng, Hsuan-Te Chu, Chia-Hung Kao

**Affiliations:** ^1^Division of Pulmonary and Critical Care Medicine, Department of Internal Medicine, Tri-Service General Hospital, National Defense Medical Center, Taipei, Taiwan; ^2^Management Office for Health Data, China Medical University Hospital, Taichung, Taiwan; ^3^College of Medicine, China Medical University, Taichung, Taiwan; ^4^Department of Otolaryngology-Head and Neck Surgery, Tri-Service General Hospital, National Defense Medical Center, Taipei, Taiwan; ^5^Graduate Institute of Medical Sciences, National Defense Medical Center, Taipei, Taiwan; ^6^Department of Psychiatry, Beitou Branch, Tri-Service General Hospital, National Defense Medical Center, Taipei, Taiwan; ^7^Graduate Institute of Biomedical Sciences and School of Medicine, College of Medicine, China Medical University, Taichung, Taiwan; ^8^Department of Nuclear Medicine and PET Center, China Medical University Hospital, Taichung, Taiwan; ^9^Department of Bioinformatics and Medical Engineering, Asia University, Taichung, Taiwan

**Keywords:** benzodiazepines, hypnotics, obstructive sleep apnea, acute respiratory failure, pneumonia

## Abstract

**Aims:** Obstructive sleep apnea (OSA) and insomnia commonly coexist; hypnotics are broadly prescribed for insomnia therapy. However, the safety of hypnotics use in OSA patients is unclear. We conducted a retrospective case-control study to investigate the risk of adverse respiratory events in hypnotics-using OSA patients.

**Methods:** We obtained data from the Taiwan National Health Insurance Database from 1996 to 2013. The case group included 216 OSA patients with newly diagnosed adverse respiratory events, including pneumonia and acute respiratory failure. The control group included OSA patients without adverse respiratory events, which was randomly frequency-matched to the case group at a 1:1 ratio according to age, gender, and index year. Hypnotics exposure included benzodiazepines (BZD) and non-benzodiazepines (non-BZD). A recent user was defined as a patient who had taken hypnotics for 1–30 days, while a long-term user was one who had taken hypnotics for 31–365 days.

**Results:** Multivariable adjusted analysis showed recent BZD use is an independent risk for adverse respiratory events (OR = 2.70; 95% CI = 1.15–6.33; *P* < 0.001). Subgroup analysis showed both recent and long-term BZD use increased the risk of acute respiratory failure compared to never BZD use (OR = 28.6; 95% CI = 5.24–156; *P* < 0.001, OR = 10.1; 95% CI = 1.51–67.7; *P* < 0.05, respectively). Neither BZD nor non-BZD use increased the risk of pneumonia in OSA patients.

**Conclusion:** BZD use might increase the risk of acute respiratory failure in OSA patients.

## Introduction

Obstructive sleep apnea (OSA) is a sleep disorder, characterized by repetitive partial or complete obstruction collapse of the upper airway, which leads to apnea and hypopnea during sleep. Frequent arousal from sleep terminates apnea and hypopnea, but may lead to non-restorative sleep and daytime sleepiness. The pathophysiology of OSA involves the upper airway anatomy, upper airway dilator muscle responsiveness, arousal threshold, and ventilatory control instability (White, 2005). OSA should be deemed to have a continuum with the prevalence of 6–17% in the general adult population and 49% in the elderly population (with the OSA severity of apnea-hypopnea index ≥ 15) (Senaratna et al., 2017). The prevalence of OSA has been also increasing in parallel with the prevalence of obesity over the past two decades (Dempsey et al., 2010; Flegal et al., 2010; Memtsoudis et al., 2013; Peppard et al., 2013). OSA has become a crucial health issue worldwide; evidences has demonstrated that phenotypes with excessive daytime sleepiness (clusters 2, 3, 4) is the independent predictor of cardiovascular diseases, metabolic disorders and all-cause mortality (Tasali and Ip, 2008; Gagnadoux et al., 2016; Garbarino et al., 2018). While most OSA patients remain undiagnosed, OSA has imposed a considerable economic burden on healthcare systems (Young et al., 1997; Tarasiuk and Reuveni, 2013).

The prevalence of insomnia in OSA patients is approximately 39–58%, and the phenotypes with higher insomnia prevalence are female OSA (cluster 1), mildly symptomatic OSA (cluster 4), and comorbid OSA (cluster 5) (Luyster et al., 2010; Gagnadoux et al., 2016). OSA patients with the comorbidity of insomnia may have increased sleep apnea severity, and the coexistence of both diseases may lead to more symptoms associated with depression, anxiety, and stress than those observed in patients with OSA alone (Smith et al., 2004). Furthermore, many OSA patients have other comorbidities including anxiety, epilepsy, and panic disorder, and take hypnotics for therapy (Hollinger et al., 2006; Rezaeitalab et al., 2014; Su et al., 2015). However, even in generalized use, the safety of hypnotics in OSA patients is inconclusive. One early study suggested that the use of hypnotics in patients with sleep apnea was associated with decreased upper airway muscle tone, and reduced ventilatory response to desaturation that led to an increased AHI (Hanly and Powles, 1993). A recent review suggested there was no deleterious effect of the hypnotics on the severity of OSA, as measured by AHI or ODI; however, most of the recruited studies were small and had a short follow-up period (Mason et al., 2015). Furthermore, although non-BZD have similar hypnotic and sedative effects, and fewer muscle-relaxant properties compared with BZD, the respiratory adverse effects of different hypnotics in OSA patients are undetermined. Therefore, we conducted a population-based case-control study to investigate the effect of hypnotics (BZD and non-BZD) on the risk of adverse respiratory events in patients with OSA.

## Materials and Methods

### Data Source

The government of Taiwan launched the NHI program in 1995, which covers more than 99% of Taiwan’s population (Wu et al., 2012). The insurance claims database is called the NHIRD. The data for this study were obtained from the LHID 2000, which is a sub-data set of the NHIRD, and randomly selects one million participants from the NHIRD. The representativeness of the LHID 2000 for all insurance enrollees has been validated (Cheng et al., 2011). The ICD-9-CM was used for disease identification in the NHIRD. The database includes patient information and insurance claims. To protect patient privacy, all identifiers were scrambled and re-encoded to strengthen data security. This study was approved by the International Review Board of the China Medical University and Hospital Research Ethics Committee (IRB permit number: CMUH104-REC2-115-CR3).

### Study Participants

**FIGURE 1:**
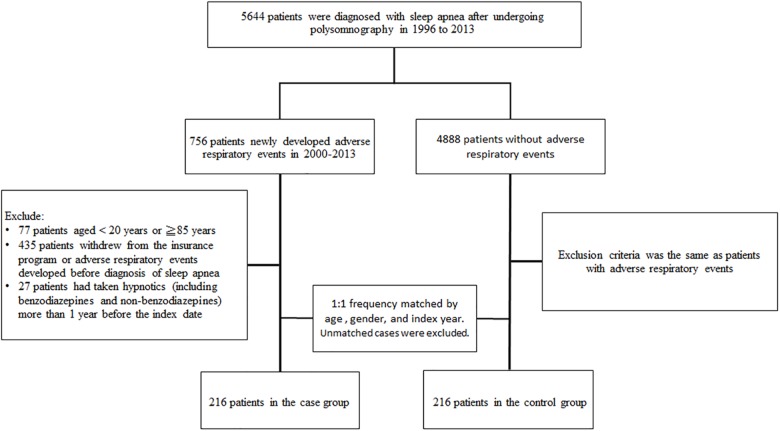
A flow chart that identifies the number of patients and study design.

A prior study externally validated the diagnosis of sleep apnea in the NHIRD cohort, and reported that nearly 99% of the cases diagnosed with sleep apnea were the obstructive type, with only 0.9% diagnosed with pure central apnea (Su et al., 2014). Thus, in this study, OSA was defined as a diagnosis of sleep apnea (ICD-9-CM codes 327.23, 780.51, 780.53, 780.57) made using an overnight polysomnography (NHI codes 17008A, 17008B) test. The case group of this study was OSA patients aged 20–85 years who were newly diagnosed with adverse respiratory events (pneumonia [ICD-9-CM codes 480–486], and acute respiratory failure [ARF, ICD-9-CM code 518.81]) between 2000 and 2013. The index date was the date on which the patients were diagnosed with adverse respiratory events. Further, we excluded patients who had a history of adverse respiratory events before the onset of OSA. The control group was randomly selected from OSA patients who had never been diagnosed with adverse respiratory events. The control group contained the same number of patients as did the case group and was frequency-matched with the case group with respect to age (every 5 years), gender, and index year. We also excluded those patients who had received hypnotics (BZDs and Non-BZDs) more than 1 year before the index date. A flowchart describing participant selection is shown in Figure 1.

### Exposure

In this study, we examined exposures to hypnotics, including BZD (flurazepam, nitrazepam, flunitrazepam, estazolam, triazolam, lormetazepam, midazolam, and brotizolam) and non-BZD (zopiclone, zolpidem, and zaleplon). Based on the period of hypnotics prescription, we classified patients into three groups. The first group was patients who had never received hypnotics before the index date. The group of recent users was patients who had received hypnotics for 1–30 days before the index date. Finally, the long-term users group consisted of patients who had received hypnotics for 31–365 days before the index date.

### Relevant Variables and Comorbidities

The CCI was calculated using the patient’s comorbidities before the index date and was weighted for each disease. The comorbidities we examined in this study were coronary artery disease (ICD-9-CM codes 410–414), congestive heart failure (ICD-9-CM codes 428, 398.91, and 402.x1), diabetes mellitus (ICD-9-CM code 250), hypertension (ICD-9-CM codes 401–405), stroke (ICD-9-CM codes 430–436), hyperlipidemia (ICD-9-CM code 272), chronic kidney disease (ICD-9-CM codes 582, 583, 585, 586, and 588), obesity (ICD-9-CM code 278.0), depression (ICD-9-CM codes 296.2, 296.3, 300.4, and 311), anxiety (ICD-9-CM code 300.00), dementia (ICD-9-CM codes 290, 294.1, and 331.0), chronic obstructive pulmonary disease (COPD, ICD-9-CM codes 491, 492, and 496), insomnia (ICD-9-CM codes 780.52), epilepsy (ICD-9-CM codes 345), and panic disorder (ICD-9-CM codes 300.01). We also considered whether the patients had received treatment for sleep apnea. Such treatments included CPAP treatment and surgery (pharyngeal or nasal surgery).

### Statistical Analysis

Table 1 shows the demographics of the case and control groups and the results of testing the differences between the two groups using a Chi-squared test for categorical variables and a *t*-test for continuous variables. We used univariate and multivariate unconditional logistic regression to estimate the OR and 95% confidence interval (CI) of adverse respiratory events in hypnotics users. The variables in the multivariate model were CCI score and the comorbidities of congestive heart failure, diabetes mellitus, hypertension, chronic kidney disease, obesity, depression, COPD, and insomnia. The data analysis for this study was performed using SAS statistical software (Version 9.4 for Windows; SAS Institute, Inc., Cary, NC, United States), and a *p*-value of less than 0.05 was considered to indicate statistical significance.

**Table 1:** Baseline characteristics of patients.

	Adverse respiratory outcomes				*p*-value
	Yes (*n* = 216)		No (*n* = 216)		
	*n*	%	*n*	%	
**Gender**					1.00
Male	160	74.1	160	74.1	
Female	56	25.9	56	25.9	
**Age**					1.00
30–49	43	19.9	43	19.9	
50–64	90	41.7	90	41.7	
65–84	83	38.4	83	38.4	
Mean (*SD*)	55.3 (15.3)		55.1 (15.3)		0.88
**Hypnotics use**					
Benzodiazepines use					0.003
Never use	176	81.5	200	92.6	
Recent use (1–30 days)	24	11.1	9	4.17	
Long-term use (31–365 days)	16	7.41	7	3.24	
Non-benzodiazepines use					0.03
Never use	145	67.1	169	78.2	
Recent use (1–30 days)	40	18.5	27	12.5	
Long-term use (31–365 days)	31	14.4	20	9.26	
**Charlson comorbidity index**					<0.0001
0	132	61.1	172	79.6	
1	31	14.4	27	12.5	
2	22	10.2	9	4.17	
≥3	31	14.4	8	3.70	
**Comorbidity**					
Coronary artery disease	98	45.4	89	41.2	0.38
Congestive heart failure	46	21.3	18	8.33	0.0001
Diabetes mellitus	54	25.0	36	16.7	0.03
Hypertension	136	63.0	105	48.6	0.003
Stroke	67	31.0	52	24.1	0.11
Hyperlipidemia	118	54.6	106	49.1	0.25
Chronic kidney disease	52	24.1	35	16.2	0.04
Obesity	21	9.72	8	3.70	0.01
Depression	40	18.5	25	11.6	0.04
Anxiety	59	27.3	51	23.6	0.38
Dementia	13	6.02	5	2.31	0.05
COPD	84	38.9	58	26.9	0.008
Insomnia	61	28.2	38	17.6	0.009
Epilepsy	11	5.09	7	3.24	0.34
Panic disorder	5	2.31	1	0.46	0.10
**Treatment status**					0.21
Without treatment	176	81.5	189	87.5	
CPAP	13	6.02	6	2.78	
Surgery	26	12.0	21	9.72	
CPAP and Surgery	1	0.46	0	0	


*COPD, chronic obstructive pulmonary disease; CPAP, continuous positive airway pressure*.

## Results

In this study, both the case and control group had 216 patients, and the proportion of males was higher than that of females. The mean age of the case group and control group were 55.3 and 55.1 years, respectively. The case group had a higher ratio of patients who used BZDs and Non-BZDs than the control group, and had higher proportions of all the comorbidities. There was no significant difference in the proportion of patients who received therapy for OSA between the case and control group.

Table 2 shows the risk factors of adverse respiratory events, and we considered them in a multivariate unconditional logistic regression model. Compared with the patients who had never received BZDs, the adjusted OR of adverse respiratory events in recent users of BZDs was 2.70 (95% CI = 1.15–6.33, *P* < 0.001). The adjusted ORs of adverse respiratory events in patients with non-BZD use were not significantly different from those in non-users. Patients with higher CCI scores had higher adjusted ORs for adverse respiratory events. With respect to comorbidities, congestive heart failure was an independent risk for adverse respiratory events (OR = 1.97, 95% CI = 1.00–3.88, *P* < 0.05). Whether OSA patients received treatment or not did not significantly affect the risk of adverse respiratory events.

**Table 2:** Risk factors of adverse respiratory events for the patients with obstructive sleep apnea.

	Crude		Adjusted	
	OR	(95%CI)	OR	(95%CI)
**Hypnotics use**				
Benzodiazepines use				
Never use	1	(Reference)	1	(Reference)
Recent use (1–30 days)	3.03	(1.37,6.69)^∗∗^	2.70	(1.15,6.33)^∗∗∗^
Long-term use (31–365 days)	2.6	(1.04,6.46)^∗^	1.80	(0.67,4.87)
Non-benzodiazepines use				
Never use	1	(Reference)	1	(Reference)
Recent use (1–30 days)	1.73	(1.01,2.95)^∗^	1.53	(0.85,2.75)
Long-term use (31–365 days)	1.81	(0.99,3.31)	0.86	(0.42,1.78)
**Gender**				
Male	1	(Reference)		
Female	1.00	(0.65,1.54)		
**Age**				
30–49	1	(Reference)		
50–64	1.00	(0.60,1.67)		
65–100	1.00	(0.59,1.68)		
**Charlson comorbidity index**				
0	1	(Reference)	1	(Reference)
1	1.5	(0.85,2.63)	1.19	(0.63,2.23)
2	3.19	(1.42,7.15)^∗∗^	1.78	(0.74,4.32)
≥3	5.05	(2.25,11.35)^∗∗∗^	3.09	(1.26,7.56)^∗^
**Comorbidity**				
Coronary artery disease	1.18	(0.81,1.73)		
Congestive heart failure	2.98	(1.66,5.33)^∗∗∗^	1.97	(1.00,3.88)^∗^
Diabetes mellitus	1.67	(1.04,2.67)^∗^	1.01	(0.59,1.73)
Hypertension	1.80	(1.22,2.64)^∗∗^	1.06	(0.66,1.69)
Stroke	1.42	(0.93,2.17)		
Hyperlipidemia	1.25	(0.86,1.82)		
Chronic kidney disease	1.64	(1.02,2.64)^∗^	0.87	(0.50,1.53)
Obesity	2.8	(1.21,6.47)^∗^	2.30	(0.93,5.70)
Depression	1.74	(1.01,2.98)^∗^	1.46	(0.80,2.66)
Anxiety	1.22	(0.79,1.88)		
Dementia	2.70	(0.95,7.72)		
COPD	1.73	(1.15,2.60)^∗∗^	1.19	(0.74,1.90)
Insomnia	1.84	(1.17,2.92)^∗∗^	1.37	(0.80,2.33)
Epilepsy	1.60	(0.61,4.21)		
Panic disorder	5.09	(0.59,44.0)		
**Treatment status**				
Without treatment	1	(Reference)		
CPAP	2.33	(0.87,6.25)		
Surgery	1.33	(0.72,2.45)		
CPAP and Surgery	–	–		


*OR, odds ratio; CI, confidence interval; CPAP, continuous positive airway pressure. Models adjusted by Charlson comorbidity index and comorbidities of congestive heart failure, diabetes mellitus, hypertension, chronic kidney disease, obesity, depression, COPD, and insomnia. ^∗^p < 0.05, ^∗∗^p < 0.01, ^∗∗∗^p < 0.001.*

In Table 3, we show a subgroup analysis of the different adverse respiratory events. Compared with the patients who had never received BZDs, the adjusted OR of pneumonia in recent users and long-term users was not statically significant. The recent use of BZDs increased the risk of acute respiratory failure with the adjusted OR of 28.6 (95% CI = 5.24–156, *P* < 0.001), and so did the long-term use of BZD with adjusted OR of 10.1 (95% CI = 1.51–67.7, *P* < 0.05). However, neither the recent nor long-term use of non-BZD significantly increased the risk of pneumonia or acute respiratory failure.

**Table 3:** The relationship between hypnotics use and the different adverse respiratory events.

		Adjusted					
	Number of cases	OR (95%CI)		OR (95%CI)		OR (95%CI)		
	Never use/recent use/long-term use	Never use		Recent use (1–30 days)		Long-term use (31–365 days)	
**Benzodiazepines use**				
Adverse respiratory events							
Pneumonia (*N* = 193)	162/19/12	1	(Reference)	2.24	(0.92,5.46)	1.62	(0.57,4.56)
Acute respiratory failure (*N* = 23)	14/5/4	1	(Reference)	28.6	(5.24,156)^∗∗∗^	10.1	(1.51,67.7)^∗^
**Non-benzodiazepines use**				
Adverse respiratory events							
Pneumonia (*N* = 193)	129/37/27	1	(Reference)	1.59	(0.88,2.87)	0.88	(0.42,1.84)
Acute respiratory failure (*N* = 23)	16/3/4	1	(Reference)	0.45	(0.05,4.37)	0.61	(0.12,3.19)


*OR, odds ratio; CI, confidence interval. Models adjusted by Charlson comorbidity index and comorbidities of congestive heart failure, diabetes mellitus, hypertension, chronic kidney disease, obesity, depression, COPD, and insomnia. ^∗^p < 0.05, ^∗∗∗^p < 0.001.*

Supplementary Table S1 showed that the crude OR of adverse respiratory events in only midazolam users was 3.79 (95% CI = 1.03–14.0, *P* < 0.05), but the adjusted OR was non-significant (95% CI = 0.83–12.8). Supplementary Table S2 showed that recent use of midazolam increased the risk of adverse respiratory events with the adjusted OR of 5.26 (95% CI = 1.07–25.8, *P* < 0.05). Supplementary Table S3 showed that 16.1% of patients with pneumonia and 39.1% of patients with acute respiratory failure ever took BZD; 3.63% of patients with pneumonia and 13.0% of patients with acute respiratory failure had recent use of midazolam.

## Discussion

To our knowledge, this is the first case-control study to investigate whether hypnotics use increases the risk of adverse respiratory events in OSA patients. Our study suggests that recent BZD use (1–30 days) increases the risk of adverse respiratory events (OR = 2.7), and subgroup analysis suggested BZD use increased the risk of acute respiratory failure. We did not find these risks in OSA patients who were non-BZD users.

An expert meeting of the Canadian Sleep Society and a review article by Guilleminault suggest that it is inappropriate to prescribe BZD for patients with OSA because of possible complete airway obstruction (Guilleminault, 1990; Hanly and Powles, 1993). Our findings strengthen this evidence, and further outline the increased risk of acute respiratory failure in this clinical setting. Similarly, Gonçalves et al. (2013) retrospectively analyzed 515 patients with OSA diagnosed by polysomnography and clinical symptoms, and found that lower minimum oxygen saturation was associated with BZD use. Combining Gonçalves’s finding and the result of the present study, we suggest that when OSA patients who use BZD encounter some precipitating factors such as fragility or infection, low oxygen saturation could exacerbate and may lead to acute respiratory failure. On the other hand, a review by Mason et al. reported that BZD, including nitrazepam, temazepam, brotizolam, flurazepam, and triazolam, did not worsen OSA as measured by AHI or RDI (Mason et al., 2015). However, the numbers of cases in the selected studies were small (no more than 20), and the use of both flurazepam 20 mg and triazolam 0.25 mg resulted in a higher AHI than did placebo administration, although the difference was not statistically significant (Cirignotta et al., 1988; Berry et al., 1995).

Some pathophysiological mechanisms of the negative effect of hypnotics on OSA have been postulated. First, hypnotics might suppress central ventilatory drive by binding BZD receptors, especially the BZ2 receptor, to activate the GABA system in motor neurons, the limbic system, and the dorsal horn of the spinal cord (Rudolf et al., 1978; Griffin et al., 2013). This respiratory depressant effect might be synergistic when BZDs are used in combination with opioids (Jones et al., 2012). Second, hypnotics might reduce muscle tone in OSA patients with already functionally morbid upper airway dilator muscles, and then further lead to increased AHI (Guilleminault, 1990; Ankichetty et al., 2011; Ejaz et al., 2011). Third, arousal from sleep induced by blood gas change with respiratory effort possibly activates the upper airway dilator muscles and reopens the upper airway; this is considered a lifesaving event in OSA patients. Hypnotics might increase the arousal threshold, which leads to delayed airway opening and the exacerbation of hypoxia and hypercapnia (Berry et al., 1995; Eckert et al., 2011; Jordan et al., 2017). Fourth, cyclic alternating pattern (CAP) in electroencephalography is the marker of a sleep instability that reflects the brain’s effort to preserve the structure of sleep (Parrino et al., 2012, 2014). The occurrence of CAP was verified to be significantly correlated with the apneas, hypopneas or flow limitation events in OSA patients (Bosi et al., 2018). BZD use in OSA patients may decrease the CAP rate in non-rapid eye movement sleep which results in less resilience to adverse respiratory events.

In the present study, the use of BZD increased the risk of acute respiratory failure in OSA patients, but non-BZD use did not. The slightly different pharmacological effects of BZD and non-BZD may account for this disparity. First, non-BZD have fewer respiratory suppression effects than BZD, although most the evidences have been obtained from studies on patients with chronic lung diseases or healthy participants (Maillard et al., 1992; Murciano et al., 1993). Second, although both BZD and non-BZD are GABA receptor agonists, they have different receptor affinity and different binding sites on the GABA receptor, which lead to the consequence that non-BZD have less prominent muscle-relaxant effects than do BZD (Mason et al., 2015). Third, according to pharmacokinetic studies, BZD could have short, intermediate, or long-acting durations, while most non-BZD are short-acting agents. Thus, the negative effects of hypnotics on respiratory systems are prominent and last longer after BZD use than after non-BZD use, (Buscemi et al., 2005) although studies on the direct comparison of non-BZD and short-acting BZD are not available. Finally, prior studies implied that BZD increases the arousal threshold in OSA patients but non-BZD did not (Berry et al., 1995; Smith et al., 2017). But these studies only included a few kinds of hypnotics, the conclusive effect on arousal threshold of BZD and non-BZD warrants further investigation.

An elaborate and large-cohort study conducted by Su et al. (2014) reported that OSA patients had a 1.2-fold higher risk of developing pneumonia compared with subjects without sleep apnea. The pathogenesis of this connection may be related to increased aspiration risk in OSA patients caused by higher negative pressure and decreased sensation in the upper airway due to an impaired swallowing reflex (Teramoto et al., 1999; Kimoff et al., 2001; Dempsey et al., 2010). Our study observed that the BZD or non-BZD use did not have a statistically significant OR for developing pneumonia, so further extrapolated that hypnotic use is not a predisposing factor for pneumonia development in OSA patients. Furthermore, a population-based and case-control study by Chen et al. (2018) reported that BZD users had a higher risk of hospitalization for pneumonia than did non-BZD users. The result of our study did not conflict with Chen’s study, because the study design differed, and our case and control groups were composed of OSA patients instead of general population. In combinative summary of Su, Chen and the present studies, (Su et al., 2014; Chen et al., 2018) OSA patients have increased risk of developing pneumonia, but hypnotics (including BZD or non-BZD) use is not a predisposing factor for pneumonia development in OSA patients.

Obstructive sleep apnea patients have an increased frequency of comorbidity with major depressive disorder, posttraumatic stress disorder and anxiety (Kjelsberg et al., 2005; Gupta and Simpson, 2015). Increased mortality and exacerbation of OSA have been reported when OSA patients were treated with psychotropic medication such as antidepressants and antipsychotics for psychiatric disorders (Linselle et al., 2017; Jennum et al., 2018). We could not exclude the possibility that the combined use of BZD and other psychotropic medication in OSA patients may have additively or synergistically negative effect on the respiratory system which could increase the risk of adverse respiratory events. However, this hypothesis needs further evidences to verify.

There were several limitations in the present study. First, although we investigated the single drugs in BZD, the adverse respiratory event risk may not be disclosed in some drugs owing to small sample size (Supplementary Table S2), which was also the reason we could not further analyze in term of dosage, potency or half-life. However, the single drugs in BZD and non-BZD may still show subtle diversity in their pharmacodynamics or pharmacokinetics, and the adverse respiratory events risk of each drug in OSA patients needs further investigation. Second, the AHI in polysomnography and the clinical symptoms of the OSA patients were not available in the NHIRD; thus, we could not subanalyze the risk of adverse respiratory events based on OSA severity. Although we tried to categorize CPAP use and surgery as severe OSA, this is not a standard classification. The CPAP compliance was also unavailable, so we could not analyze whether CPAP therapy protect the negative effect of BZD use. Third, disease identification in the NHIRD was performed according to the ICD-9-CM, so coding mistakes by physicians were possible. However, the Taiwan National Health Insurance authorities frequently reviews the medical records to inspect the accuracy of the ICD coding, and the OSA and pneumonia coding accuracy in the NHIRD has been externally validated by prior studies (Shiao et al., 2013; Su et al., 2014). Fourth, there are unpreventable biases in any retrospective study, even though we performed an adjusted analysis for potential confounders. Fifth, for retrospective study design according to registry codes, this study could not show if BZD impaired sleep apnea or if sleep apnea impairment was associated with increased risk of adverse respiratory events. Finally, almost all the patients recruited in this study were Taiwanese; the extrapolation of this results to other ethnicities needs further validation.

## Conclusion

Our study suggested that BZD use in OSA patients increased the risk of acute respiratory failure. Prior study reported OSA patients had increased risk of developing pneumonia, the present study further extrapolated that neither BZD nor non-BZD use is a predisposing factor for pneumonia development in OSA patients. However, OSA severity classification and individual drug dosage analysis could not be performed in the present study that may generate biases in the conclusion. Further studies with more specific designs are needed to investigate the safety between individual hypnotics and diverse OSA phenotypes.

## Author Contributions

All authors contributed significantly and are in agreement with the content of the manuscript. S-HW and C-HK conceived and designed the study. C-HK provided the study materials. S-HW, W-SC, S-ET, H-CL, C-KP, H-TC, and C-HK collected and assembled the data, analyzed and interpreted the data, wrote the manuscript, and approved the final manuscript.

## Conflict of Interest Statement

The authors declare that the research was conducted in the absence of any commercial or financial relationships that could be construed as a potential conflict of interest.

## Abbreviations

AHI: apnea hypopnea index
BZD: benzodiazepines
CCI: Charlson comorbidity index
CI: confidence interval
CPAP: continuous positive airway pressure
GABA: gamma amino butyric acid
ICD-9-CM: International Classification of Diseases, Ninth Revision, Clinical Modification
LHID2000: Longitudinal Health Insurance Database 2000
NHI: National Health Insurance
NHIRD: National Health Insurance Research Database
Non-BZD: non-benzodiazepines
ODI: oxygen desaturation index
OR: odds ratio
OSA: obstructive sleep apnea
