# Supplementary material for: Benzodiazepines Associated With Acute Respiratory Failure in Patients With Obstructive Sleep Apnea
Source: Front Pharmacol. 2019 Jan 7;9:1513. doi: 10.3389/fphar.2018.01513 (PMC6330300; doi:10.3389/fphar.2018.01513)
Supplement: Supplementary file 1 [file Table_1.DOC]

| **STable 1. Odds ratios of adverse respiratory events for obstructive sleep apnea patients with Benzodiazepines use** | | | | | | | | |
| --- | --- | --- | --- | --- | --- | --- | --- | --- |
|  | **Adverse respiratory event** | |  | **Crude** | |  | **Adjusted** | |
|  | **Yes** | **No** | | **OR** | **(95%CI)** |  | **OR** | **(95%CI)** |
|  | **n (%)** | **n (%)** |  |  |  |  |  |  |
| **Hypnotics use** |  |  |  |  |  |  |  |  |
| **Benzodiazepines use** |  |  |  |  |  |  |  |  |
| Never use | 176 (81.5) | 200 (92.6) |  | 1 | (Reference) |  | 1 | (Reference) |
| Only flurazepam | 2 (0.93) | 0 (0) |  | - | - |  | - | - |
| Only nitrazepam | 2 (0.93) | 1 (0.46) |  | 2.27 | (0.20,25.3) |  | 2.53 | (0.18,36.5) |
| Only flunitrazepam | 2 (0.93) | 0 (0) |  | - | - |  | - | - |
| Only estazolam | 13 (6.02) | 8 (3.70) |  | 1.85 | (0.75,4.56) |  | 1.47 | (0.54,3.96) |
| Only triazolam | 5 (2.31) | 1 (0.46) |  | 5.68 | (0.66,49.1) |  | 5.30 | (0.57,48.8) |
| Only lormetazepam | 1 (0.46) | 0 (0) |  | - | - |  | - | - |
| Only midazolam | 10 (4.63) | 3 (1.39) |  | 3.79 | (1.03,14.0)* |  | 3.26 | (0.83,12.8) |
| Only brotizolam | 1 (0.46) | 2 (0.93) |  | 0.57 | (0.05,6.32) |  | 0.71 | (0.06,8.44) |
| Combination use | 4 (1.85) | 1 (0.46) |  | 4.55 | (0.50,41.0) |  | 2.02 | (0.19,21.9) |
| OR, odds ratio; CI, confidence interval | | | | | | | | |
| Models adjusted by Charlson comorbidity index and comorbidities of congestive heart failure, diabetes mellitus, hypertension, chronic kidney disease, obesity, depression, COPD, and insomnia. | | | | | | | | |
| *p<0.05, **p<0.01, ***p<0.001 | | | | | | | | |

| **STable 2. Odds ratios of adverse respiratory events for obstructive sleep apnea patients with recent or long-term Benzodiazepines use** | | | | | |
| --- | --- | --- | --- | --- | --- |
|  | **Adverse respiratory event** | |  | **Adjusted** | |
|  | **Yes** | **No** | | **OR** | **(95%CI)** |
|  | **n (%)** | **n (%)** |  |  |  |
| **Hypnotics use** |  |  |  |  |  |
| **Benzodiazepines use** |  |  |  |  |  |
| Never use | 176 (81.5) | 200 (92.6) |  | 1 | (Reference) |
| Only flurazepam |  |  |  |  |  |
| Recent use (1-30 days) | 1 (0.46) | 0 (0) |  | - | - |
| Long-term use (31-365 days) | 1 (0.46) | 0 (0) |  | - | - |
| Only nitrazepam |  |  |  |  |  |
| Recent use (1-30 days) | 1 (0.46) | 1 (0.46) |  | 0.85 | (0.03,22.6) |
| Long-term use (31-365 days) | 1 (0.46) | 0 (0) |  | - | - |
| Only flunitrazepam |  |  |  |  |  |
| Recent use (1-30 days) | 2 (0.93) | 0 (0) |  | - | - |
| Long-term use (31-365 days) | 0 (0) | 0 (0) |  | - | - |
| Only estazolam |  |  |  |  |  |
| Recent use (1-30 days) | 5 (2.31) | 4 (1.85) |  | 1.14 | (0.26,4.94) |
| Long-term use (31-365 days) | 8 (3.70) | 4 (1.85) |  | 1.76 | (0.48,6.42) |
| Only triazolam |  |  |  |  |  |
| Recent use (1-30 days) | 3 (1.39) | 1 (0.46) |  | 3.57 | (0.35,36.8) |
| Long-term use (31-365 days) | 2 (0.39) | 0 (0) |  | - | - |
| Only lormetazepam |  |  |  |  |  |
| Recent use (1-30 days) | 1 (0.46) | 0 (0) |  | - | - |
| Long-term use (31-365 days) | 0 (0) | 0 (0) |  | - | - |
| Only midazolam |  |  |  |  |  |
| Recent use (1-30 days) | 10 (4.63) | 2 (0.93) |  | 5.26 | (1.07,25.8)* |
| Long-term use (31-365 days) | 0 (0) | 1 (0.46) |  | - | - |
| Only brotizolam |  |  |  |  |  |
| Recent use (1-30 days) | 0 (0) | 1 (0.46) |  | - | - |
| Long-term use (31-365 days) | 1 (0.46) | 1 (0.46) |  | 1.47 | (0.08,25.8) |
| Combination use |  |  |  |  |  |
| Recent use (1-30 days) | 1 (0.46) | 0 (0) |  | - | - |
| Long-term use (31-365 days) | 3 (1.39) | 1 (0.46) |  | 1.54 | (0.13,18.5) |
| OR, odds ratio; CI, confidence interval | | | | | |
| Models adjusted by Charlson comorbidity index and comorbidities of congestive heart failure, diabetes mellitus, hypertension, chronic kidney disease, obesity, depression, COPD, and insomnia. | | | | | |
| *p<0.05, **p<0.01, ***p<0.001 | | | | | |

| **STable 3. Number of adverse respiratory events for obstructive sleep apnea patients with recent or long-term Benzodiazepines use** | | | |
| --- | --- | --- | --- |
|  | **Adverse respiratory event** | | |
|  | **Yes** | | **No** |
|  | **Pneumonia** | **Acute respiratory failure** |  |
|  | **n (%)** | **n (%)** | **n (%)** |
| **Hypnotics use** |  |  |  |
| **Benzodiazepines use** |  |  |  |
| Never use | 162 (83.9) | 14 (60.9) | 200 (92.6) |
| Only flurazepam |  |  |  |
| Recent use (1-30 days) | 1 (0.52) | 0 (0) | 0 (0) |
| Long-term use (31-365 days) | 1 (0.52) | 0 (0) | 0 (0) |
| Only nitrazepam |  |  |  |
| Recent use (1-30 days) | 1 (0.52) | 0 (0) | 1 (0.46) |
| Long-term use (31-365 days) | 1 (0.52) | 0 (0) | 0 (0) |
| Only flunitrazepam |  |  |  |
| Recent use (1-30 days) | 2 (1.04) | 0 (0) | 0 (0) |
| Long-term use (31-365 days) | 0 (0) | 0 (0) | 0 (0) |
| Only estazolam |  |  |  |
| Recent use (1-30 days) | 5 (2.59) | 0 (0) | 4 (1.85) |
| Long-term use (31-365 days) | 6 (3.11) | 2 (8.70) | 4 (1.85) |
| Only triazolam |  |  |  |
| Recent use (1-30 days) | 2 (1.04) | 1 (4.35) | 1 (0.46) |
| Long-term use (31-365 days) | 1 (0.52) | 1 (4.35) | 0 (0) |
| Only lormetazepam |  |  |  |
| Recent use (1-30 days) | 1 (0.52) | 0 (0) | 0 (0) |
| Long-term use (31-365 days) | 0 (0) | 0 (0) | 0 (0) |
| Only midazolam |  |  |  |
| Recent use (1-30 days) | 7 (3.63) | 3 (13.0) | 2 (0.93) |
| Long-term use (31-365 days) | 0 (0) | 0 (0) | 1 (0.46) |
| Only brotizolam |  |  |  |
| Recent use (1-30 days) | 0 (0) | 0 (0) | 1 (0.46) |
| Long-term use (31-365 days) | 1 (0.52) | 0 (0) | 1 (0.46) |
| Combination use |  |  |  |
| Recent use (1-30 days) | 0 (0) | 1 (4.35) | 0 (0) |
| Long-term use (31-365 days) | 2 (1.04) | 1 (4.35) | 1 (0.46) |
|  | | | |
